# Changing Patterns of SARS-CoV-2 Seroprevalence: A Snapshot among the General Population in Kuwait

**DOI:** 10.3390/vaccines11020336

**Published:** 2023-02-02

**Authors:** Wadha Alfouzan, Haya Altawalah, Ahmad AlSarraf, Walid Alali, Talal Al-Fadalah, Fahad Al-Ghimlas, Saud Alajmi, Mubarak Alajmi, Ebtehal AlRoomi, Ahlam Jeragh, Rita Dhar

**Affiliations:** 1 Department of Microbiology, Faculty of Medicine, Kuwait University, Kuwait City 46300, Kuwait; 2 Microbiology Unit, Department of Laboratory Medicine, Farwania Hospital, Ministry of Health, Kuwait City 85000, Kuwait; 3 Virology Unit, Department of Laboratory Medicine, Kuwait Cancer Control Center, Ministry of Health, Kuwait City 20001, Kuwait; 4 Biochemitry Unit, Department of Laboratory Medicine, Kuwait Cancer Control Center, Ministry of Health, Kuwait City 20001, Kuwait; 5 Department of Epidemiology and Biostatistics, Faculty of Public Health, Kuwait University, Kuwait City 13110, Kuwait; 6 Quality and Accreditation Directorate, Ministry of Health, Kuwait City 13001, Kuwait; 7 Public Health Directorate, Ministry of Health, Kuwait City 20001, Kuwait; 8 Ahmadi Hospital, Administration Chief Clinical Services and Chief Supportive Clinical Services, Kuwait City 13126, Kuwait; 9 Microbiology Unit, Department of Laboratory Medicine, Jahra Hospital, Ministry of Health, Jahra 00020, Kuwait; 10 Microbiology Unit, Department of Laboratory Medicine, Adan Hospital, Ministry of Health, Kuwait City 46969, Kuwait

**Keywords:** SARS-CoV-2, hybrid immunity, Kuwait, general population, COVID-19 vaccination

## Abstract

We sought to assess pre-vaccination and post-vaccination seroprevalences of anti-SARS-CoV-2 antibodies in Kuwait and to compare antibody levels between vaccine types. In phase 1 (pre-vaccination period, *n* = 19,363), blood samples were collected before the launch of COVID-19 vaccination in Kuwait between 1 September and 31 December 2020. Blood samples for phase 2 (post-vaccination period, *n* = 4973) were collected between 1 September and 30 November 2021. We tested subjects for anti-SARS-CoV-2 antibodies using the DiaSorin LIAISON^®^ SARS-CoV-2 IgM and Trimeric S IgG tests. In the pre-vaccination period, the prevalence of SARS-CoV-2 IgM and IgG was 14.50% (95% CI: 14.01–15.00) and 24.89% (95% CI: 24.29–25.50), respectively. The trend of seropositivity increased with age and was higher for females and non-Kuwaiti participants (*p* < 0.0001). Interestingly, seroprevalence was significantly higher for those who had received one dose of BNT162b2 (95.21%) than those who had received one dose of ChAdOx1-nCov-19 (92.86%). In addition, those who reported receiving two doses had higher seroprevalence, 96.25%, 95.86%, and 94.93% for ChA-dOx1-nCov-19/AstraZeneca, mix-and-match, and BNT162b2 recipients, respectively. After the second dose, median spike-specific responses showed no significant difference between ChAdOx1-nCov-19 and BNT162b2. Furthermore, statistical analysis showed no significant difference between median anti-trimeric S antibody levels of vaccinated individuals according to sex, age, or nationality (*p* > 0.05). In contrast, a negative correlation between age and anti-trimeric S IgG titers of BNT162b2-vaccinated subjects was observed (r = −0.062, *p* = 0.0009). Antibody levels decreased with time after vaccination with both vaccines. Our findings indicate that seroprevalence was very low during the pre-vaccination period (25%) in the general population and was greater than 95% in the vaccinated population in Kuwait. Furthermore, ChAdOx1-nCov-19 and BNT162b2 are effective in generating a similar humoral response.

## 1. Introduction

Coronaviruses are positive-sense, single-stranded RNA viruses of the family that are naturally present in a number of birds and mammals. The novel coronavirus (CoV) that emerged from Wuhan of Hubei province in China has spread to several countries worldwide, including Kuwait. It was found to be highly homologous to severe acute respiratory syndrome coronavirus (SARS-CoV), which caused severe acute respiratory syndrome (SARS) in thousands of people in 2003 [1]. The SARS-CoV-2, now associated with the coronavirus disease 2019 (COVID-19) might also be transmitted from bats and cause similar symptoms through a similar mechanism to that observed with SARS-CoV [2]. Since the initial epidemic of COVID-19 has evolved into a pandemic, there has been debate about how to halt the rapid spread of this disease [3]. As of 28 October 2022, the COVID-19 pandemic caused by the SARS-CoV-2 virus had caused more than 626,337,158 cases and 6,566,610 deaths worldwide, and 12,830,378,906 vaccine doses had been administered (https://covid19.who.int, accessed 28 October 2022). In Kuwait, from 3 January 2020 to 28 October 2022, there were 661,787 confirmed cases of COVID-19 with 2566 deaths, and a total of 8,227,208 vaccine doses was administered (https://covid19.who.int/region/emro/country/kw, accessed 28 October 2022).

The detection and profile of specific antibodies to SARS-CoV-2 can provide valuable information for rapid screening of suspected cases, assist in diagnosis, and importantly, evaluate the disease course even after recovery. Thus, understanding the protective capacity and the duration of humoral immunity during SARS-CoV-2 infection or after vaccination is critical for managing the pandemic and will also provide more evidence about the efficacy of SARS-CoV-2 vaccines [4]. Additionally, identification of SARS-CoV-2 seroprevalence can provide estimates of community transmission of viral infection and vaccine coverage that can be used as additional surveillance tools as the pandemic progresses.

Several seroprevalence studies have been conducted throughout the world. According to the global SARS-CoV-2 seroprevalence dashboard (https://serotracker.com/, assessed on 27 June 2022), a total of 3465 seroprevalence studies have been reported in 137 countries and territories with 29,448,001 participants [5]. In Kuwait, an early survey conducted at the beginning of the pandemic (between 18 April and 10 May 2020) estimated overall seroprevalence (IgG or IgM) at 5.9%, indicating low percentages of seropositivity [6].

The two doses of COVID-9 SARS-CoV-2 vaccine are very effective in preventing symptomatic SARS-CoV-2 infection, the incidence of which is somewhat declined six months after full vaccination [7,8]. The rollout of COVID-19 vaccine in Kuwait began on 24 December 2020, following high efficacy demonstrated in a randomized placebo-controlled trial [4]. The two COVID-19 vaccines currently available in Kuwait are Pfizer-BioNTech and AstraZeneca-Oxford have been licensed for all persons aged ≥12 years at the time of the study. Analyses of antibody response to COVID-19 vaccine and robustness of seroconversion have not yet been examined in Kuwait although data presented by other investigators show distinct results [9,10,11,12]. Furthermore, the durability of protection, especially with the emergence of variants of concern, is limited in time [10,13]. In addition, the response to vaccination may vary depending on prior exposure to SARS-CoV-2 and the genetic background of the population [5,12]. Thus, the prevalence of IgG antibodies to SARS-CoV-2 in a population should reflect the prevalence of recovered and vaccinated cases with likely effective sustained immunity. Moreover, seroprevalence estimates are essential to describing the immune landscape of SARS-CoV-2 and to guiding public health decisions [14].

To the best of our knowledge, this is the first study that has attempted to describe pre-vaccine seroprevalence (1 September to 31 December 2020) and post-vaccine seroprevalence (1 September to 30 November 2021) in the general population of Kuwait with 75% vaccine coverage (https://ourworldindata.org/covid-vaccinations?country=KWT, accessed 28 October 2022). Moreover, we quantified the post-vaccination humoral immune response based on the types of vaccines deployed by the Ministry of Health in Kuwait. Finally, we examined the humoral response over time. The results of this investigation are expected to assist decision makers at the Ministry of Health in developing policies to control the epidemic and in making recommendations regarding the use of the fourth dose of COVID-19 vaccine.

## 2. Materials and Methods

### 2.1. Ethical Considerations

The study was approved by the permanent Committee for Coordination of Medical and Health Research, Ministry of health, Kuwait (no. 1469/2020) and the study was conducted in accordance with the ethical guidelines of the 1975 Declaration of Helsinki as reflected in a priori approval by the institution’s human research committee. Oral or written informed consent was obtained from all participants and parents of children under 15 years of age.

### 2.2. Study Design and Population

The study was designed as a cross-sectional survey in the general population and surveys were conducted according to our sampling plan (two phases):

Phase 1: Remaining blood (whole blood or serum) samples taken upon physician request (a convenience non-random collection), for any reason, at five general hospitals across Kuwait between was our target population. The blood samples represented a diverse range of patients (outpatient and inpatient) belonging to both genders and five age groups. The blood samples (total *n* = 19,363) were collected before launching the COVID-19 vaccination in Kuwait from 1 September to 31 December 2020 (pre-vaccination period) and provided demographic information and a blood donation. The estimated minimum sample size was based on an expected 10% positive IgM/IgG with 95% CI and 2% desired precision around the estimate and resulted in about 1000 participants per stratum per month. The selection of blood samples from the existing samples was done using a weighted random sampling method at each hospital.

Phase 2: Immune status in vaccinated subjects was evaluated. Blood specimens (*n* = 4973) collected after the initiation of the COVID-19 vaccination campaign in Kuwait, from 1 September 2021 to 30 November 2021, were assessed for the level of seroprevalence of SARS-CoV-2 in order to assess the target population immunity and also to investigate the antibody level according to demographic information. A stratified random sampling method to select subjects was used. The strata were based on the vaccine status (first dose only, two doses, or three doses) and type of vaccine (Pfizer or non-Pfizer). The three-dose group was included as one group (due to small number of subjects) without dividing it into two groups based on vaccine type. As such, using a total of five strata, subjects were selected from each stratum using a weighted random sampling method.

### 2.3. Data Collection

During the first three months of the project, a designated technician at each hospital collected a specific number of leftover blood samples each week according to our sampling plan (stratified random sampling based on a weekly sampling frame of all samples received during that week). The selected blood samples were stored and sent for antibody analysis at the virology laboratory at the Faculty of Medicine (Kuwait University).

During the second months of the post-vaccination project, an assigned technician at each hospital collected a specific number of remaining blood samples each week in accordance with our sampling plan (stratified random sampling based on a weekly sampling frame of all samples received during that week). Age, sex, and nationality data were available with the samples. Selected blood samples were stored and sent for antibody analysis to the Virology Laboratory of the Faculty of Medicine (Kuwait University).

### 2.4. Biological Sample Collection, Handling, Transport, and Testing 

At least 1 mL of plasma/serum was collected from each patient. Blood samples collected from the hospitals were centrifuged in the laboratory; the serum/plasma was stored at 4 °C until transported in a cold icebox to the Virology/FOM with the patient’s demographics data. Plasma/serum samples were transferred to a newly labeled 1.8 mL tube and then placed in a box at −20 °C until tested (up to 3 months). Two separate sheets were created in Microsoft Excel. In the first sheet, all samples collected during phase 1 were gathered (*n* = 19,363) and in the second sheet, samples collected during phase 2 (*n* = 4973).

### 2.5. Detection of SARS-CoV-2 Antibodies

We collected a baseline blood sample (2–3 mL) from all the study participants to test for the presence of total SARS-CoV-2 antibodies. Combined detection of IgM and IgG is used to assess the immune status of individuals exposed and infected with SARS-CoV-2. To estimate the seroprevalence IgG against the SARS-CoV-2 infection before vaccination (Phase 1), plasma samples were tested using the automated Liaison SARS-CoV-2 on LIAISON^®^ XL (DiaSorin S.p.A, Saluggia, Italy). The DiaSorin LIAISON^®^ SARS-CoV-2 IgM, was used to detect IgM antibodies to SARS-CoV-2 with a specificity of 99.2% according to the manufacturer’s assessment. The results were analyzed and interpreted according to the manufacturer’s instructions. In addition, plasma samples were tested using the LIAISON^®^SARS-CoV-2 Trimeric S IgG assay, a chemiluminescent immunoassay for the quantitative determination of anti-trimeric spike-protein-specific IgG antibodies to SARS-CoV-2 with clinical sensitivity of 98.7% and clinical specificity of 99.5%. The analyzer automatically calculates the SARS-CoV-2 IgG antibody levels expressed as BAU/mL and grades the results. Patient results should be interpreted as follows: BBAU/mL < 33.8 as a negative, BAU/mL ≥ 33.8 as a positive.

To estimate the level of S-protein-binding IgG in the vaccinated groups with and without previous documented natural exposure (Phase 2), we used the LIAISON^®^SARS-CoV-2 Trimeric S IgG assay. The test showed good correlation with the microneutralization test and yielded a percent positive agreement (PPA) of 100% and a percent negative agreement (NPA) of 96.9% based on the manufacturer’s evaluation.

### 2.6. Statistical Analysis

Continuous variables were reported as medians with interquartile ranges (IQR), while categorical variables were reported as percentages. The relationship between the seroprevalence and potential characteristics (sex, age groups, home location by governorate, and nationality) was assessed using 2 × 2 chi-square. Binomial exact 95% confidence intervals (CIs) were constructed around proportions. Continuous variables were compared using Mann–Whitney and Kruskal–Wallis tests. Statistical analyses were performed using STATA software version 15.1 (STAT Corp., College Station, TX, USA) and GraphPad PRISM version 6.0e (GraphPad Software, San Diego, CA, USA). All *p*-values were two-sided and a *p*-value of less than 0.05 was used as a cut-off value for statistical significance.

## 3. Results

### 3.1. Seroprevalence in the General Population during the Pre-Vaccination Period Due to Natural Infection

To better understand the level of exposure to the SARS-CoV-2 in Kuwait, we conducted a cross-sectional study from 1 September 2020 to 31 December 2020 (pre-vaccination period). A total of 19,453 serum samples was collected from the general population and analyzed for SARS-CoV-2 IgM and IgG. The prevalence of SARS-CoV-2 IgM was 14.50% (95% CI: 14.01–15.00).

The prevalence of SARS-CoV-2 IgG was 24.89% (95% CI: 24.29–25.50%). In univariate models, higher prevalence of anti-S positivity was associated with sex, age, and nationality of participants (all comparisons *p* < 0.0001). In fact, seroprevalence was significantly higher in women (31.1%) than in men (28.4%) (*p* < 0.0001) (Table 1). Furthermore, after adjustment for variables in the multivariate logistic regression model, participants in the 45–64 age group were significantly more likely more likely to be seropositive than the reference group (<15 years) (adjusted OR = 1.4, *p* < 0.0001). Interestingly, analysis by nationality showed that Kuwaiti participants had a significantly lower probability of being seropositive compared with non-Kuwaiti citizens (Table 1).

### 3.2. Seroprevalence in the General Population during the Post-Vaccination Period

Compared with seroprevalence in unvaccinated adults, seroprevalence was significantly higher for those who received one dose of BNT162b2/Pfizer (95.21%) than for those who received ChAdOx1-nCov-19/AstraZeneca (92.86%) (Table 2). Seroprevalence was higher among two-dose recipients at 96.25%, 95.86%, and 94.93% for ChAdOx1-nCov-19/AstraZeneca, AstraZeneca-Pfizer, and BNT162b2/Pfizer, respectively (Table 2).

### 3.3. Antibody Response to One, Two, and Three Doses of SARS-CoV-2 Vaccine in the General Population of Kuwait

**Figure 1:**
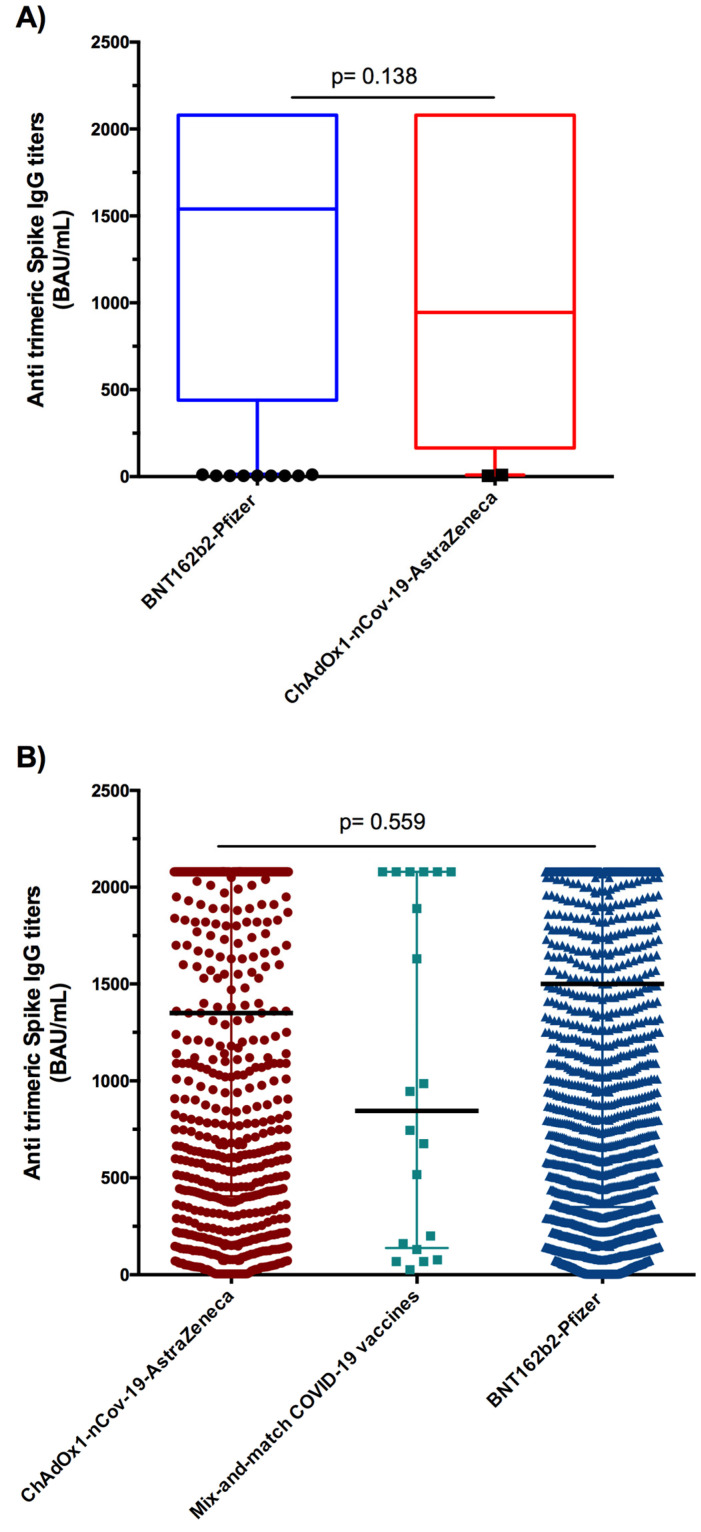
Severe acute respiratory syndrome coronavirus 2 (SARS-CoV-2) anti-trimeric S antibody response to COVID-19 vaccination in the general population. (**A**) Comparison of antibody responses between ChAdOx1-nCoV-19/AstraZeneca and BioNTech162b2/Pfizer vaccines after two doses. (**B**) Anti-trimeric S antibody levels in initially seronegative subjects after two doses of ChA-dOx1-nCoV-19/AstraZeneca, mix-and-match, and BioNTech162b2/Pfizer vaccines. Data are presented as median and interquartile range for IgG antibody titers. Mann–Whitney and Kruskal–Wallis tests were used for comparisons.

We then assessed the magnitude of the humoral response using the SARS-CoV-2 trimeric spike IgG assay. Median anti-trimeric S antibody concentrations were 944.5 BAU/mL and 1540 BAU/mL after one dose of ChAdOx1-nCov-19/AstraZeneca and BNT162b2-Pfizer, respectively. No significant difference was observed between AstraZeneca and Pfizer vaccines (Figure 1A). After the second dose, the median spike-specific responses showed no significant difference between the vaccines (Figure 1B).

**Figure 2:**
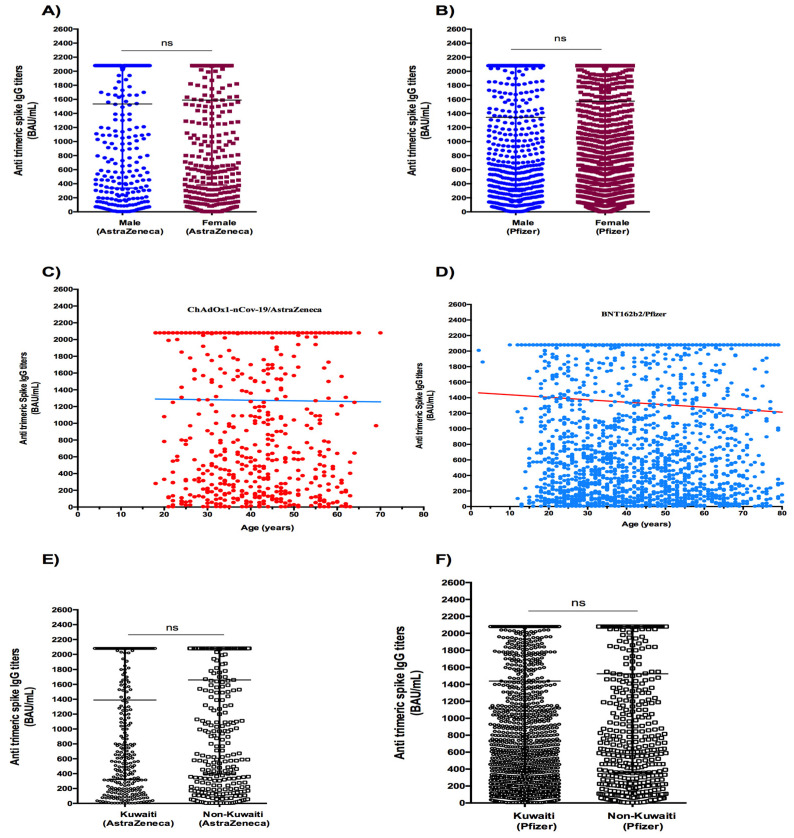
Antibody responses to SARS-CoV-2 S protein after two doses of ChAdOx1-nCoV-19/AstraZeneca and BioNTech162b2/Pfizer vaccines. Antibody titers by gender after ChAdOx1-nCoV-19/AstraZeneca (**A**) and BioN-Tech162b2/Pfizer (**B**) vaccines. Correlation of age and anti-SARS-CoV-2 antibody levels according to age after ChAdOx1-nCoV-19/AstraZeneca (**C**) and BioNTech162b2/Pfizer vaccine (**D**). Antibody titers according to ethnicity after ChAdOx1-nCoV-19/AstraZeneca (**E**) and BioNTech162b2/Pfizer vaccine (**F**). Data are presented as median and interquartile range for IgG antibody. Spearman correlation, Mann–Whitney and Kruskal–Wallis tests were used.

No significant difference in titers by gender was observed in subjects who received two doses of ChAdOx1-nCov-19/AstraZeneca vaccine (median = 1535 vs. 1590 BAU/mL for males and females, respectively) (*p* = 0.535) (Figure 2A) and BNT162b2/Pfizer vaccine (1345 vs. 1575 BAU/mL for males and females, respectively) (*p* = 0.188) (Figure 2B). No correlation was found between age and spike IgG levels in participants who received two doses of ChAdOx1-nCov-19/AstraZeneca vaccine (r = −0.004, 95% CI: -0.07 to 0.07, *p* = 0.899) (Figure 2C). In contrast, Spearman’s bivariate analysis showed a negative correlation between age and anti-trimeric spike IgG titers in subjects vaccinated with BNT162b2-Pfizer (r = −0.062, 95% CI: −0.100 to −0.025, *p* = 0.0009) (Figure 2D). No significant difference in titers by ethnicity was observed in subjects receiving two doses of ChAdOx1-nCov/19-AstraZeneca (median = 1390 vs. 1660 BAU/mL for Kuwaitis and non-Kuwaitis, respectively) (*p* = 0.780) (Figure 2E), and BNT162b2/Pfizer vaccine (median = 1440 vs. 1525 BAU/mL for Kuwaitis and non-Kuwaitis, respectively) (*p* = 0.236) (Figure 2F).

**Figure 3:**
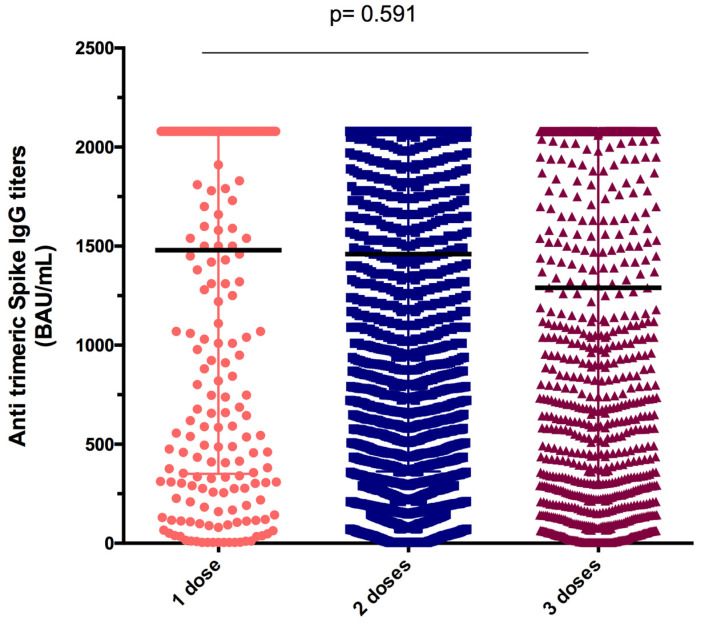
Antibody titers after one, two, and three doses of COVID-19 vaccine. Data are presented as median and interquartile range for IgG antibody titers. Kruskal–Wallis test was used for comparisons.

Finally, comparison between anti-trimeric S antibody concentrations in samples after the one dose (median = 1480 BAU/mL), two doses (median = 1460 BAU/mL), and three doses (median 1290 BAU/mL) showed no statistical difference between vaccines (*p* = 0.591) (Figure 3).

**Figure 4:**
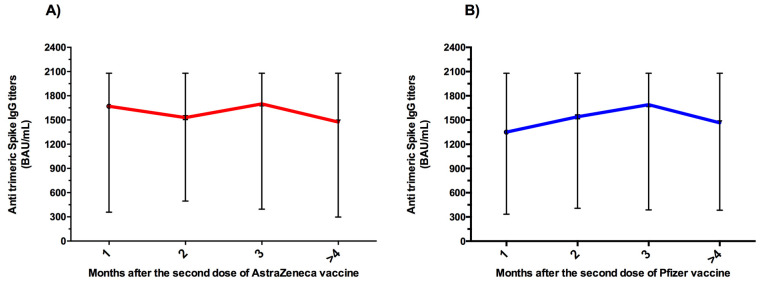
Anti-trimeric S antibody levels according to time since the second dose of ChAdOx1-nCoV-19/AstraZeneca (**A**) and BioNTech162b2/Pfizer vaccine (**B**). Data are presented as median and interquartile range for IgG antibody titers.

In participants who received the full vaccination, anti-trimeric S antibody concentrations one month after the second dose were highest with ChAdOx1-nCov-19/AstraZeneca (median = 1670 BAU/mL), followed by BNT162b2/Pfizer (median = 1350 BAU/mL). In this study, after 3 months, median antibody titers decreased for both vaccines without reaching significance (*p* > 0.05) (Figure 4). Indeed, after the second dose of SARS-CoV-2 vaccination, the median anti-S antibody titer at 4 months (median = 1475 BAU/mL) was decreased compared with the titer at 3 months (median = 1700 BAU/mL) in those vaccinated with ChAdOx1-nCov-19/AstraZeneca (Figure 4A). In addition, the median titer of trimeric spike IgG in the Pfizer-vaccinated group was highest 3 months after vaccination (median = 1690 BAU/mL) but began to decline after 4 months (median = 1465 BAU/mL) (Figure 4B).

**Figure 5:**
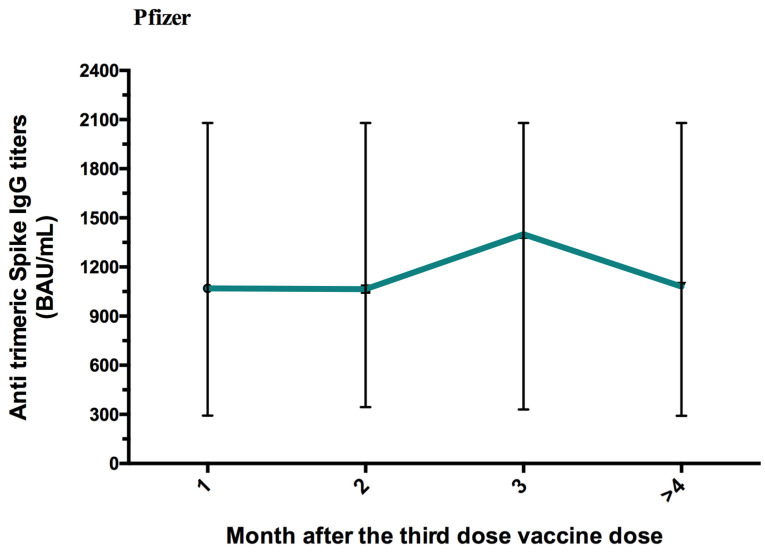
Anti-trimeric S antibody titers according to time from third dose of BioNTech162b2/Pfizer vaccine.

In boosted-vaccinated participants (received three doses of Pfizer vaccine), anti-trimeric S antibody concentrations peaked at 3 months after the third dose and then decreased (Figure 5). However, we did not analyze the antibody levels in participants vaccinated with three doses of AstraZeneca vaccine because the number of participants was very small (*n* = 6).

## 4. Discussion

Serum antibody testing is the most robust investigation for assessing prior exposure to SARS-CoV-2 infection because it can detect both symptomatic and asymptomatic cases [15,16]. Similar to our previous first-round survey from April to May 2020 [6], our current study involved multiple governorates in Kuwait and estimated active infection by assessing IgM and post-exposure infection using IgG antibodies. In the first round of seroprevalence studies, the adjusted overall seroprevalence of SARS-CoV-2 (IgM/IgG) was 5.9% at the beginning of the pandemic [6]. In this study, IgM seroprevalence was 14.76%, higher than previously reported in the first wave and similar to that reported in Iran (14%) during the same period [17], highlighting an active circulation of SASR-CoV-2 during the study period in Kuwait. In addition, the estimated prevalence of IgG in the pre-vaccination period (25%) was remarkably higher than the first report [6]. This demonstrates an increase in seropositivity in Kuwait with the progression of the pandemic. Our seroprevalence rates are similar to those reported in neighboring countries such as Saudi Arabia (24.3%) [18], Oman (22%) [19], and Iran (23.8%) [20]. This could be because in late 2020, rapid increases in SARS-CoV-2 infection rates were observed in all age groups with the emergence of the more transmissible Alpha variant. However, this prevalence was low compared to the seroprevalence reported in Qatar (64.4%) between 21 June and 9 September 2020 [21], and higher than that reported in unvaccinated individuals in Germany (1.3–2.8%) between July and December 2020 [22]. Thus, the observed difference between countries was more likely driven by the epidemic conditions and health regulations applied in each region [23].

In our study, seroprevalence was higher in females (31%) than males (28%). However, conflicting data have been reported regarding gender and IgG responses to SARS-CoV-2 infection. Similar results have been observed in previous seroprevalence studies, which reported higher seroprevalence in women than in men [24,25,26]. In contrast, other serological surveys have shown no association between SARS-CoV-2 IgG prevalence and gender [27,28,29,30].

Significant differences were found between age groups, mainly in the 45–64 age group compared to the 15–24 age group. These results are consistent with other investigations that have shown that adolescents and the elderly have lower seroprevalence rates than other age groups [19,28,29,31]. In contrast, previous surveys have identified high seroprevalence in younger age groups, which may be due to the continued operation of schools and universities throughout the epidemic [32,33,34,35].

The rate of seropositivity was higher among non-Kuwaitis than among Kuwaitis. This result is similar to other serological surveys conducted in Gulf Cooperation Council countries [19,21,28,36] and could be attributed to the confined work camps, housing conditions with shared accommodation, and poor adherence to social distance to avoid SARS-CoV-2 infection [19,36]. No significant differences in humoral responses between individuals who received ChAdOx1-nCoV-19/AstraZeneca and BioNTech162b2/Pfizer after two doses of vaccine were found, which appears to be consistent with previous studies [37,38]. Nevertheless, it appears that the ChA-dOx1-nCoV-19/AstraZeneca vaccine may induce higher levels of specific T cells, whereas mRNA vaccines may induce higher antibody titers [39,40].

To our knowledge, this is the first study to attempt to describe antibody seroprevalence in the general population of Kuwait after deployment of the first, second, and third doses of COVID-19 vaccines. Overall, we found a 95% prevalence of previously vaccinated participants who developed IgG. These data are consistent with seroconversion rates previously observed in published reports [37,41,42]. For both vaccines, we noted that IgG titers decreased over time after vaccination. These results appear to be in agreement with previous studies showing a continuous decrease in antibody titers over time, particularly in susceptible individuals [43,44,45,46,47,48]. However, further evaluation of other immune-system effector mechanisms, including non-neutralizing antibodies, T cells, and innate immunity mechanisms, is needed to understand both the immune response after vaccination and how to limit the severity of SARS-CoV-2, especially in vulnerable populations (elderly and immunocompromised subjects), who often have inadequate antibody responses [49,50].

Our survey had several limitations. The study sample was not representative of the general population, which may have resulted in selection bias, and it is likely that seroprevalence data during the pre-vaccination period are underestimated. This selection bias should be clearly accounted for in future studies Another limitation is that we were not able to evaluate antibody neutralization tests and cellular immunity, given our limited resources.

## 5. Conclusions

To our knowledge, there are, to date, no other published studies estimating the seroprevalence of SARS-CoV-2 in a large group in 2021 in Kuwait. These serological survey results indicate that seroprevalence was 25% during the pre-vaccination period and higher than that reported during the initial phase of the pandemic (18 April–10 May 2020). The increase in seroprevalence between May 2020 and December 2020 reflects the magnitude of the second wave of the pandemic. Interestingly, more than 95% of the general population generated an immune response to SARS-CoV-2 in Kuwait after vaccination. The antibody response rate to vaccination is comforting, however further studies are needed to correlate the antibody response with functional immunity, to estimate the duration of protection from infection, and to determine the effect of the fourth dose of vaccine on the duration of vaccine effectiveness, in particular with the ongoing emergence of variants of concern variants of SARS-CoV-2.

## Figures and Tables

**Table 1:** Seroprevalence estimates stratified by sex, age, and nationality in Kuwait from 1 September 2020 to 31 December 2020.

Characteristics	No. Participants	Seroprevalence (%) (95% CI)	*p*-Value	Adjusted OR (95% CI)	*p*-Value
Sex *					
Male	2189	28.4 (27.4–29.5)	<0.0001	1.00	
Female	2769	31.1 (30.1–32.0)		1.3 (1.2–1.4)	<0.0001
Age group (years) *			<0.0001		
<15	328	23.0 (20.9–25.3)		1.00	
15–24	530	20.5 (19.0–22.1)		1.1 (1–1.3)	0.06
25–44	1736	26.0 (24.9–27.0)		1.2 (1.0–1.3)	0.02
45–64	1619	30.0 (28.4–30.8)		1.4 (1.2–1.6)	<0.0001
≥65	742	23.1 (21.7–24.6)		1.0 (0.9–1.2)	0.940
Nationality *			<0.0001		
Kuwaiti	2305	20.8% (20.0–21.6)		1.00	
Non-Kuwaiti	2629	32.2% (31.2–33.2)		1.8 (1.7–1.9)	<0.0001

* data are missing.

**Table 2:** SARS-CoV-2 seroprevalence in general population in Kuwait during post-vaccination period, 1 September 2021 to 30 November 2021.

COVID-19 Vaccination Status	General Population
**1 dose** BNT162b2 Pfizer -IgG positive, (%) (95% CI) ChAdOx1-nCov-19 AstraZeneca-IgG positive, (%) (95% CI) **2 doses** BNT162b2 Pfizer -IgG positive, (%) (95% CI) ChAdOx1-nCov-19 AstraZeneca-IgG positive, (%) (95% CI) Mix-and-match AstraZeneca- Pfizer IgG positive, (%) (95% CI) **3 doses** IgG positive, (%) (95% CI)	95.21 (91.15–97.46) 92.86 (80.99–97.54) 94.93 (94.06–95.68) 96.25 (94.67–97.38) 95.86 (76.39–99.11) 94.67 (93.18–95.85)

## Data Availability

The corresponding author had full access to all the data in the study.
